# Transcriptome Analysis Reveals the Requirement of the TGFβ Pathway in Ascidian Tail Regression

**DOI:** 10.3390/cells14070546

**Published:** 2025-04-04

**Authors:** Wenjie Shi, Penghui Liu, Dongyu Yang, Yuan Zhuang, Boyan Lin, Bo Dong

**Affiliations:** 1 Fang Zongxi Center for Marine EvoDevo, MoE Key Laboratory of Marine Genetics and Breeding, College of Marine Life Sciences, Ocean University of China, Qingdao 266003, China; wenjie.shi0713@stu.ouc.edu.cn (W.S.); liupenghui@stu.ouc.edu.cn (P.L.); yangdyu123456@163.com (D.Y.); zhuangyuanzty1313@163.com (Y.Z.); linboyan@stu.ouc.edu.cn (B.L.); 2 Laboratory for Marine Biology and Biotechnology, Qingdao National Laboratory for Marine Science and Technology, Qingdao 266237, China; 3 Institute of Evolution & Marine Biodiversity, Ocean University of China, Qingdao 266003, China

**Keywords:** TGFβ pathway, ascidian, metamorphosis, tail regression

## Abstract

Metamorphosis is a common developmental process in invertebrate development. It is essential for the degeneration of larval organs, formation of adult organs, and adaptation transformation of the living environment. However, the underlying molecular regulatory mechanism remains to be elucidated. In this study, we used tail regression of ascidian *Styela clava* as a model to understand the gene regulation pathway and molecular mechanism in organ metamorphosis. The TGFβ signaling pathway was screened and demonstrated to be involved in tail regression based on RNA sequencing on the different larval stages and verification with inhibitor treatment experiments. We further investigated the downstream gene network of the TGFβ signaling pathway through comparative transcriptome data analysis on the TGFβ pathway inhibition samples. Together with qRT-PCR verification, we identified four critical gene functional categories, including ion transporters/water channel, extracellular matrix structural constituent, extracellular matrix organization, and cell polarity establishment. Furthermore, a cross-species comparative analysis between *Ciona robusta* and *S. clava* was performed to understand the conservation and divergence of gene regulation in ascidians. Overall, our work identifies a crucial gene regulation pathway in ascidian tail regression and provides several potential downstream targets for understanding the molecular mechanism of larval metamorphosis.

## 1. Introduction

Metamorphosis refers to the process of post-embryonic development of an organism including birth transformation or hatching, during which significant and rapid changes in morphological structure and living habits occur between the larval and adult stages through cell proliferation and differentiation [1,2]. Metamorphosis is widely observed in the animal kingdom, including in cnidarians, mollusks, arthropods, tunicates, and some vertebrates [3,4]. Ascidian is also known as tunicates, and its special evolutionary status between invertebrates and vertebrates plays a crucial role in the study of the origin of vertebrates and the evolution of chordates [5]. The vertebrate-like ascidian larva experiences complex metamorphosis and transforms into an invertebrate-like adult. This unique process named retrograde metamorphosis can be divided into ten biological events [6]. Tail regression is the most representative process in retrograde metamorphosis, during which the notochord tissue degenerates and disappears; meanwhile, the neural tube structure disassembles and further degenerates into ganglia. In the case of the model ascidian *Ciona robusta* (also known as *C. intestinalis* Type A), the mechanism of initiating and maintaining tail regression has just been preliminarily revealed. One direction focused on searching for the signaling in initiating tail regression. The known factors are Mitogen-activated protein kinase (MAPK) signaling in inducing cell apoptosis [7,8] and GABA-GnRH axis in regulating tail regression [9]. Another focused on the mechanical mechanism. Studies have shown that actomyosin contractility inhibiton by cytochalasin B will cause failure of tail regression [10]. Detailed morphological observation found that the tail epidermis will shorten and gather at the tail posterior region, and the ROCK-dependent phosphorylation of the myosin regulatory light chain (MRLC) is necessary for epidermis shortening and the subsequent invagination by apical constriction, meanwhile, the internal tissues will enter into the trunk by transforming into coils that are not ROCK-dependent, and both mechanisms contribute to accomplishing tail regression [11]. However, the molecular regulatory mechanism in initiating tail regression and regulating highly coordinated cellular processes are still largely unexplored.

*Styela clava* is an emerging model organism in ascidian [12]. Because of its global environment adaptation [13,14] and high tolerance to temperature perturbation [15], *S. clava* shows high invasiveness and has been listed as an invasive species [16,17]; thus, understanding the molecular mechanism of larva tail regression is essential for commercial and environmental damage prevention. The genome of *S. clava* has been sequenced, the key transcription factors have been investigated [18,19]. The standard developmental atlas has been reported, the 3D reconstruction confocal images are available, and transgenic tools have been successfully applied in *S. clava* [12]. The embryogenesis process of *S. clava* is much faster and simpler compared to *C. robusta*, it takes only about 15–16 h at 18 °C to develop into a hatching larva with 22 stages based on morphology and developmental events, while *C. robusta* needs 17.5 h with 26 stages [12,20]. After hatching, *S. clava* spends around 4–5 h swimming to find a suitable shelter and then takes only about 15–30 min to adhere and regress compared to 6.5 h of swimming and 5 h of adhesion and tail regression in *C. robusta* [21]. This rapid developmental character might help explain the reason for its high invasiveness. The developmental process of *S. clava* is simpler, with no lumen in notochord tissue and without palps, and it also shows a completely different cellular behavior during tail regression [12]. However, the regulatory upstream signaling and downstream molecular mechanism in controlling rapid tail regression in *S. clava* are unclear.

In this study, we investigated the gene expression profiles in three different developmental stages of *S. clava*, namely hatched swimming larvae (hsl, which is before tail regression), tail-regressed larvae (trl, during or just finished tail regression), and metamorphic juveniles (mj, after tail regression). The functional analysis of differentially expressed genes (DEGs) was comprehensively described with the gene ontology (GO) analysis, and regulatory signal transduction pathway candidates were analyzed with the Kyoto Encyclopedia of Genes and Genomes (KEGG) analysis. We locked into the TGFβ signaling pathway and proved its necessity through the SB431542 treatment experiment. Based on the comparative transcriptome analysis between wild-type and inhibitor treatment groups, the GO enrichment analysis shows that the ion transport/channel activity, extracellular region, and lipid biosynthesis/transport processes are mainly affected. The expression levels of the top DEGs were confirmed by a qRT-PCR analysis. Ultimately, a multi-species comparison was performed to understand the conservation and divergence of gene regulatory patterns of tail regression in ascidians. These findings provide potential molecular regulatory targets to understand the rapid and highly coordinated cellular processes in ascidian tail regression.

## 2. Materials and Methods

### 2.1. Animal Collection and Fertilization

*Styela clava* and *Ciona robusta* adults were collected from the Yellow Sea in Weihai, China (122.41° E, 37.16° N) and cultured at 18 °C in a continuous circulating seawater culture system in Fang Zongxi Center, Ocean University of China. The animals were cultured for two to three days until mature gametes were produced. The healthy animals were disserted manually, and eggs and sperm were isolated from gonadal and sperm ducts, respectively. A drop of 1 mol/L Tris pH 9.5 [22] was added to activate the sperm, and the eggs and sperm were mixed to obtain fertilized eggs. After fertilization, embryos were cultured at 18 °C. The embryos were then used for RNA sequencing for the different developmental stages of wild-type samples and inhibitor treatment samples.

### 2.2. Drug Treatment and IMAGING

The *S. clava* larvae were treated with 10, 20, or 40 μM of the TGFβ signaling pathway inhibitor (SB431542, 616464, Sigma-Aldrich, St. Louis, MO, USA) [23] and DMSO at zero hours post-hatching (hph), and cultured at room temperature (23 °C) for morphological observation and imaging. Stilled images and time-lapse imaging movies were taken using differential interference contrast (DIC) microscopy (IX73 Inverted Microscope, Olympus Corporation, Tokyo, Japan). The room temperature was maintained at 23 °C by an air conditioner and the collection interval for time-lapse imaging is 2 s.

### 2.3. RNA Extraction and Transcriptome Sequencing

The total RNA of the *S. clava* inhibitor treatment samples and *C. robusta* wild-type samples was extracted with RNAiso Plus (PrimeScript™ RT reagent Kit (Perfect Real Time) (Code No. 9108), Takara Bio Inc., Kusatsu, Shiga, Japan). The integrity and quality of the total RNA were evaluated by agarose gel electrophoresis and Nanodrop spectrophotometry (NanoDrop™ OneC Microvolume UV-Vis Spectrophotometer, Thermo Fisher Scientific, Waltham, MA, USA). The library construction and sequencing experiment were accomplished by the BMKgene experimental department. mRNA was enriched and the library was constructed with NEBNext^®^ Ultra™ RNA Library Prep Kit for Illumina^®^. The library was sequenced on an Illumina Novaseq platform and 150 bp paired-end reads were generated. Clean reads were obtained from BMKgene.

### 2.4. Transcriptome Analysis

The expression profiles of different developmental stages of wild-type *S. clava* were downloaded from EDomics (http://edomics.qnlm.ac, accessed on 14 August 2024) [24], including both count and TPM data. Differential gene expression between different time points was analyzed using the DESeq2 software (accessed on 14 August 2024) [25]. Genes with a p-adjust value lower than 0.05 and a log2FoldChange greater than 2 or less than -2 were defined as differentially expressed genes (DEGs). The DEGs were visualized using a volcano plot and heatmap, which were generated by ggplot2 (version 3.5.1, accessed on 15 August 2024) [26] and pheatmap (https://CRAN.R-project.org/package=pheatmap, version 1.0.12, accessed on 15 August 2024), respectively. The gene enrichment analysis was performed using the clusterProfiler (version 4.14.6, accessed on 15 August 2024) [27] package.

Clean reads of *C. robusta* RNA-seq and *S. clava* inhibition/control groups RNA-seq were aligned against the reference genome with hisat2 [28]. The results were produced in the SAM format. Samtools [29] were used in bam file transferring and bam file sorting. Based on the sorted bam file, String Tie [30] was used in the read assembly and quantitation of transcripts with the ballgown flag activated. The TPM results were transferred based on the count file. Differentially expressed genes and gene enrichment were analyzed by the same methods.

### 2.5. Quantitative Real-Time PCR (qRT-PCR) and Analysis

qRT-PCR was used to verify the expression level of DEGs in the transcriptome results. The cDNA was synthesized by reverse transcription from the total RNA of the *S. clava* SB431542 treatment samples (HiScript II Q RT SuperMix for qPCR, Vazyme, R223-01). The primers for qRT-PCR were designed utilizing the NCBI primer design tool (https://www.ncbi.nlm.nih.gov/tools/primer-blast/, accessed on 28 October 2024) (Table 1). If more than one transcript isoforms of the selected genes were detected, the specific DNA fragment for quantifying gene expression levels was designed in the common region of all the transcript isoforms to ensure that the quantification results represent the expression levels of all the transcripts. The uniqueness of all the target fragments was verified against the *S. clava* transcriptome. RT-qPCR was performed using the SYBR Green PCR Master Mix (ChamQ Universal SYBR qPCR Master Mix (2×) (Cat# Q411-03), Vazyme Biotech Co., Ltd., Nanjing, Jiangsu, China) on Light Cycler 480 (LightCycler® 480 Real-Time PCR System, Roche Diagnostics GmbH, Mannheim, Germany). β-actin was used as the reference gene. Data were calculated using the 2^−ΔΔCt^ method. The visualization of the qRT-PCR results and the comparison with TPM in transcriptomic data were carried out with a custom-made MATLAB code (MATLAB R2024b, The MathWorks, Inc., Natick, MA, USA, accessed on 19 November 2024). All the qRT-PCR primers are listed in Table 1.

### 2.6. Cross-Species Analysis Between S. clava and C. robusta

First, the protein sequences of *S. clava* and *C. robusta* were compared using the BLAST software (version 2.15.0 accessed on 13 December 2024) [31] (blastp) to identify one-to-one homologous genes, which were then selected to form the gene set. Next, based on the TPM files of *S. clava* and *C. robusta*, the RNentropy [32] software’s RN_calc function was used to calculate the entropy values for each gene at different time points. Genes with an entropy value greater than 1 at each time point, indicating significant expression, were selected. The number of homologous genes was then determined based on the homologous gene set and used as a measure of similarity. A Sankey diagram depicting the similarity was generated using an online web tool. Homologous genes were selected from the differentially expressed genes between hatched swimming larvae vs. tail-regressed larvae and 27 hpf vs. 29 hpf, forming a gene set associated with shared pathways. The enrichment analysis and heatmap visualization were performed in the same manner.

### 2.7. Statistics

The statistical analyses were performed using custom-made MATLAB code. The number of embryo batch (biological repeat) analyzed in the experiments are indicated in the figure legends, and no statistical test was used to pre-determine sample size. The statistical distribution of each experimental group was first analyzed using the D’Agostino–Pearson normality test, then a two-sided Student’s *t*-test or a Mann–Whitney U-test was used, depending on whether the data set shows a normal distribution, *p* < 0.05 (*) indicates statistical significance.

## 3. Results

### 3.1. Morphological Observation and Gene Expression Profile of S. clava Tail Regression

**Figure 1:**
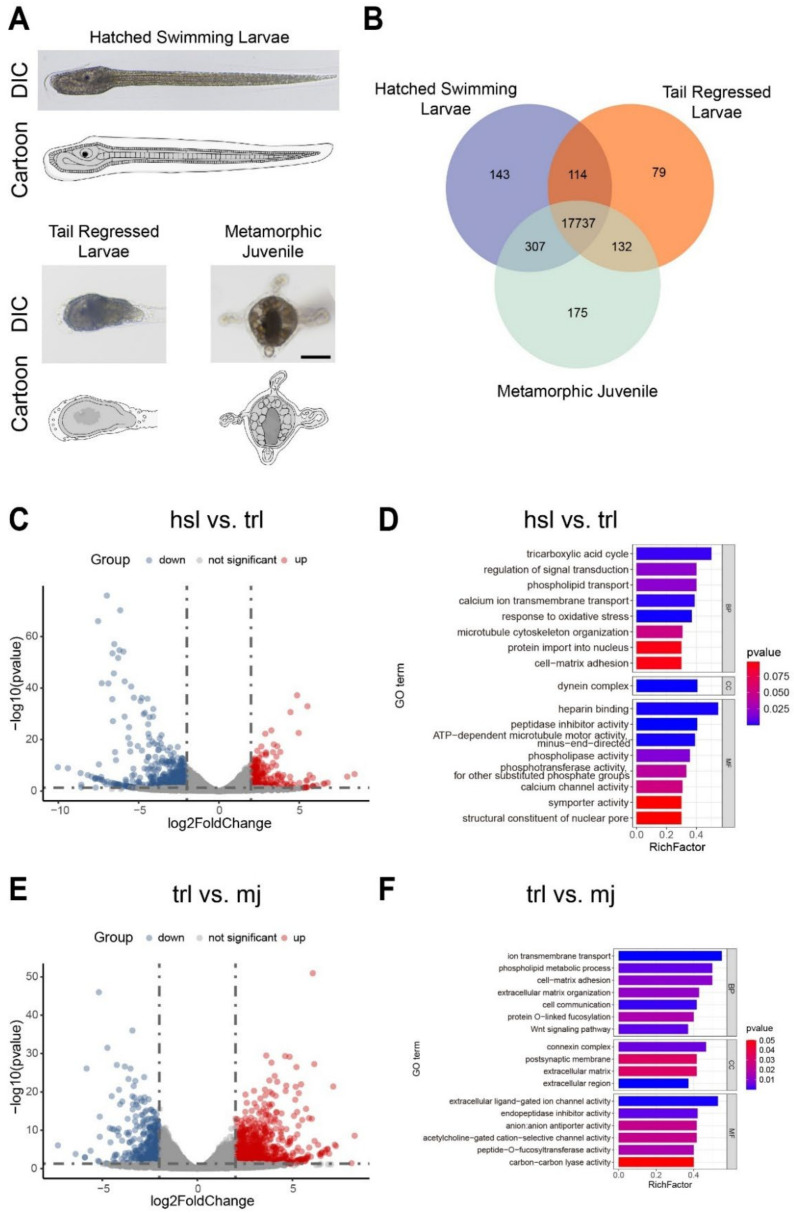
Morphology and transcriptional profile of *S. clava* at three developmental stages of tail regression: (**A**) Differential interference contrast (DIC) images (top) and cartoon (bottom) of *S. clava* in hatched swimming larva (hsl), tail-regressed larva (trl), and metamorphic juvenile (mj) stages. Scale bar, 100 μm. (**B**) Venn diagram showing the shared and unique expressed genes in hsl, trl, and mj of *S. clava*, respectively. (**C**,**E**) Volcano map of differentially expressed genes (DEGs) in the comparison of hsl vs. trl (**C**) and trl vs. mj (**E**). The red, blue, and gray dots indicate up-regulated genes, down-regulated genes, and genes with no significant expression change, respectively. The threshold was set: padj < 0.05 and |log2FC| ≥ 2. padj, adjust *p*-value, FC, fold change. (**D**,**F**) Top 20 functional items analyzed by gene ontology (GO) enrichment analysis of DEGs and sorted by RichFactor in comparison of hsl vs. trl (**D**) and trl vs. mj (**F**). BP, Biological Process; CC, Cellular Component; MF, Molecular Function.

The *S. clava* tail regression is a rapid process with drastic morphological changes. It takes only around 15–30 min to transfer from tadpole-like swimming larvae into tailless sessile-living larvae, then further develop into juveniles through metamorphosis [12]. The hatched swimming larvae have relatively clear and regular organizational boundaries and structures, with observable trunk and tail parts (Figure 1A, left). However, as the tail regression progresses, the regular organizational structure is disrupted, only the epidermis surrounding the larva is retained while the trunk sensory organ and tail tissues mix into inner cell mass (Figure 1A, middle). To comprehensively uncover the gene expression regulation during *S. clava* tail regression, we collected 0 hph (hours post-hatching, representing hatched swimming larvae) and 3 hph (representing tail-regressed larvae) with three biological repeats for RNA-seq. Additionally, considering the relatively rapid developmental period of tail regression in *S. clava*, the post-metamorphic juvenile stage was included in the transcriptome analysis to avoid the possibility of insignificant changes in gene expression patterns over a short time frame and to assist in identifying key regulatory mechanisms involved in the regression process (Figure 1A, right).

The RNA-seq data reveal significant differential gene expression at all three stages (Figure S1). The Venn diagram showed that 143, 79, and 175 genes were specifically expressed at the hatched swimming larva, tail-regressed larva, and post-metamorphic juvenile stages, respectively. Differential analysis between the three stages indicated that 185 genes were significantly up-regulated, while 416 genes were significantly down-regulated from the hatched swimming larva to the tail-regressed larva stage (Figure 1B). 637 genes were significantly up-regulated, while 324 genes were significantly down-regulated between the tail-regressed larva and the post-metamorphic juvenile stage (Figure 1D). These results highlight distinct gene expression patterns during two critical stages of metamorphosis, showing different regulatory states at each phase.

To further investigate the functions of DEGs, we performed GO enrichment analysis on these genes at various stages (Supplemental Table S1). The results showed significant differences in the pathways between the two processes. Genes at the stage prior to tail-regressed larvae were primarily enriched in the tricarboxylic acid cycle, regulation of signal transduction, phospholipid transport, ATP-dependent microtubule motor activity, minus-end-directed activity, and dynein complex (Figure 1C). In contrast, genes at the later stages were enriched in ion transmembrane transport, phospholipid metabolic process, connexin complex, phosphotransferase activity, and extracellular matrix (Figure 1E). These findings suggest that during the stage prior to tail-regressed larvae, most of the genes are involved in cell movement and migration, while in the post-metamorphic juvenile stage, more genes participate in energy metabolism and material exchange.

Next, we organized the GO pathways, and the results showed that most genes were concentrated in four categories: phospholipid metabolism, signal transduction, cytoskeleton reconstruction, and extracellular matrix, with the majority of genes being significantly down-regulated during the transition from hatched swimming larvae to tail-regressed larvae. The down-regulation of dynein heavy chain and extracellular matrix-related genes may affect the maintenance of the cytoskeleton, which could be a key change associated with tail cell regression. The up-regulation of cytosolic phospholipase A2 and phosphatidylinositol transfer protein alpha suggested that the cell membrane might undergo dissolution and remodeling during the tail regression process (Figure S2A). During the transition from tail-regressed larvae to post-metamorphic juveniles, the up-regulation of the ADAMTS family may indicate cell remodeling, while the increase in ADGRL3 could suggest enhanced intercellular adhesion. In this stage, the remodeling of the extracellular matrix appeared particularly important. The appearance of Clavanin-A may imply that the ascidian’s inner column organ is beginning to take shape, as the inner column is associated with immune functions [33].

### 3.2. Screening of Potential Regulatory Signaling Pathways During Tail Regression

To further investigate the key gene regulation profile in tail regression, we performed KEGG enrichment analysis on DEGs of two neighbor developmental stages (Supplemental Table S2). Meanwhile, to find out the upstream gene regulation mechanism rather than the downstream cellular process, we divided the KEGG enrichment results into six sub-categories: environmental information processing, cellular processes, genetic information processing, related human diseases, metabolism, and organismal systems based on the Pathway Maps (https://www.genome.jp/kegg/pathway.html, accessed on 20 August 2024). The environmental information processing sub-category mainly includes the pathways related to how cells sense and respond to external environmental signals. It covers the mechanisms by which cells perceive external stimuli (such as chemical, physical, and biological signals) and their subsequent responses, including signal transduction pathways which can help identify the upstream gene regulation pathway.

**Figure 2:**
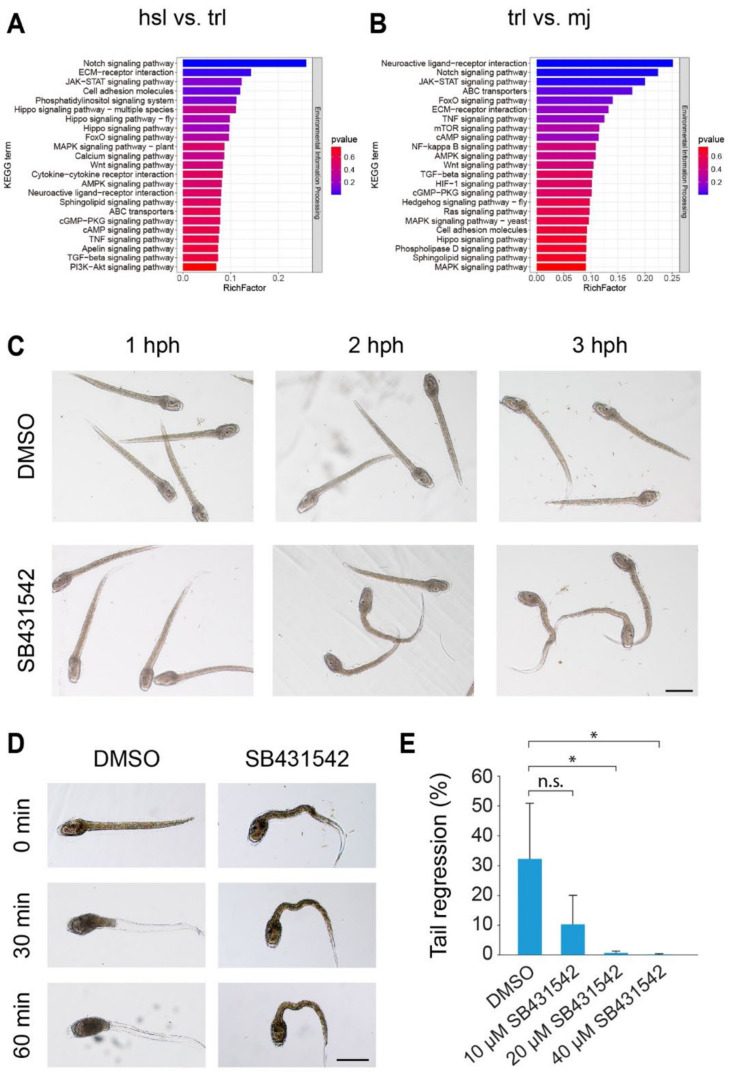
KEGG pathway annotation analysis and SB431542 treatment experiment indicate the function of the TGFβ signaling pathway in tail regression. (**A**,**B**) Potential signal transduction pathways analysis by the KEGG pathway enrichment of the DEGs in hsl vs. trl (**A**) and trl vs. mj (**B**). (**C**) DIC images showing the effect of the DMSO treatment (top) and 40 μM SB431542 (TGFβ signaling pathway inhibitor) treatment (bottom) on larva development before tail regression. The DMSO and SB431542 treatments were started at 0 hph. hph, hours post-hatching. (**D**) Time-lapse images showing the effect of the DMSO treatment (left) and 40 μM SB431542 treatment (right) on tail regression. (**E**) Quantification of the proportion of tail regression with the DMSO and SB431542 treatments (N = 3 embryo batches). Mann–Whitney *U*-test and Student’s *t*-test are performed, depending on whether data show normality distribution. *, *p* < 0.05; n.s., no significant difference. Scale bars, 100 μm.

The environmental information processing sub-category enrichment analysis showed that many potential signal transduction pathways were successfully enriched and the results were visualized by bar chart (Figure 2A, hsl vs. trl; Figure 2B, trl vs. mj), while the remaining five sub-categories were listed in Figure S3 for reference. For example, the Notch signaling pathway was at the top of the two pathway lists, it has been reported to regulate *C. robusta* embryogenesis and adult regeneration [34,35]. Meanwhile, some other signaling pathways were also enriched by the DEGs in both hsl vs. trl and trl vs. mj, and were reported to regulate *C. robusta* embryogenesis or metamorphosis, including the MAPK signaling pathway [7], TGFβ signaling pathway [36], FoxO signaling pathway [37], and Wnt signaling pathway [38].

### 3.3. Inhibition of TGFβ Signaling Pathway Cause Failure of Tail Regression

In order to confirm whether these potential KEGG-enriched and manually selected signaling transduction pathways are truly involved in the *S.clava* tail regression process, we performed signaling pathway inhibitor experiments to verify their biological function. We searched for and screened out inhibitors for the selected signaling pathways, including DAPT (Notch pathway, a γ-secretase inhibitor), U0126 (MAPK pathway, a MEK non-ATP competitive inhibitor), SB431542 (TGFβ pathway, a Smad2 phosphorylation and ALK activity inhibitor), AS1842856 (FoxO pathway, inhibit Foxo1 transcriptional activity), and XAV939 (Wnt pathway, increase β-catenin degradation through Tankyrase inhibition). These inhibitors were used to treat *S.clava* larvae and detect the phenotype on tail regression. Considering that inhibitor treatments require cumulative effects and the inhibition of certain transcription factors takes time to affect the expression levels of target genes, the phenotypes may exhibit a delayed response. Thus, the larvae were treated with inhibitors or DMSO at 0 hph for around three hours and then the influence on tail regression was detected at around 3 hph.

By using the strategy mentioned above, we performed an inhibitor concentration gradient experiment. The result illustrated that the inhibition of the Notch signaling pathway, MAPK signaling pathway, and Wnt signaling pathway have no observable phenotype; the inhibition of the FoxO signaling pathway has a slight phenotype but with no significant difference (Figure S4); and the inhibition of the TGFβ signaling pathway can cause several tail regression failure (Figure 2C–E). Based on sequential morphological observation at different time points before tail regression initiation, we found the tail part became more and more twisted within larva development (Figure 2C), indicating that the morphogenetic abnormality started at the pre-tail regression stage. After tail regression initiation, we performed high-magnification time-lapse photography, and the result showed that the larva in the inhibitor treatment group had no observable tail shortening in one hour, while most larvae in the control group could finish tail regression in the same time window (Figure 2D). The quantitive statistics showed a dose-dependent effect on the proportion of tail regression, and 20 μM SB431542 treatment could lead to almost 100% failure in tail regression. Taken together, the TGFβ signaling pathway is necessary for *S.clava* tail regression, while TGFβ pathway inhibition caused abnormality at the swimming larva stage and further led to tail regression failure.

### 3.4. Transcriptomic Profiling Reveals the Downstream Regulatory Patterns of TGFβ Pathway

After using experiments to verify the significance of the TGFβ signaling pathway for *S.clava* tail regression, we next performed RNA-seq and comparative analysis for the TGFβ pathway inhibition group and control group samples to further understand the downstream gene regulation patterns in tail regression. To collect inhibitor and DMSO treatment RNA samples, we first selected 200–250 active, morphologically normal larvae to new dishes, and then added 40 μM SB431542 (the concentration that can cause relatively several tail regression failure phenotype) or DMSO into each dish, respectively. The larvae were cultured at 23 °C from light till most larvae in the DMSO treatment group had initiated the tail regression process. After confirming that the phenotype was successfully repeated by morphological observation, the larva samples were collected. Four biological repeats were collected for RNA extraction and sequencing (Figure S5). A principal component analysis (PCA) showed that the four inhibitor treatment samples and four control samples were gathered into two main groups (Figure S6A). The correlation matrix of the eight samples also revealed high correlationship in different biological repeats (correlation index > 0.95 for most cases) and relatively low correlationship between the experiment group and control group (correlation index < 0.85 for most cases, Figure S6B). The similarity identities indicate that the inhibitor treatment exhibits good reproducibility, and the influence of the inhibitor treatment on larval gene expression patterns is much greater than the differences between different experimental batches, which gives fundamental information on SB431542 treatment on gene transcriptional regulation.

**Figure 3:**
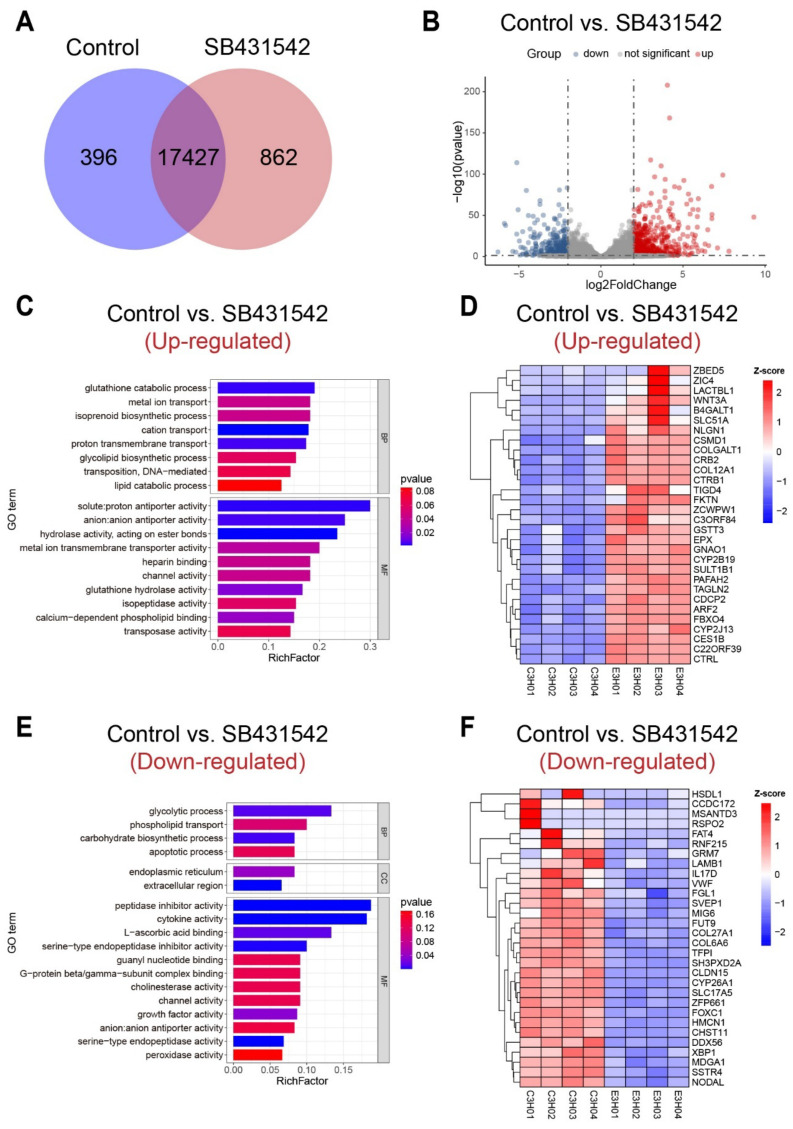
Transcriptional profile of DEGs after TGFβ signaling pathway inhibition in *S. clava* tail regression. (**A**) Venn diagram showing the shared and unique expressed genes in the control group and SB431542 treatment group. (**B**) Volcano map of DEGs in control vs. SB431542. The threshold was set the same in Figure 1. (**C**,**E**) GO enrichment analysis of the up-regulated DEGs (**C**) and down-regulated DEGs (**E**). (**D**,**F**) Heat map diagram of the top up-regulated DEGs (**D**) and top down-regulated DEGs (**F**).

Based on the gene expression profile of SB431542/DMSO treatment larvae at the tail regression stage, DEGs were detected with a filtering scale of Log2FoldChange larger than 1 and p-adjust not larger than 0.05 (Supplemental Table S3). In total, 396 and 862 genes were specifically expressed in the control group and experiment group, respectively (Figure 3A). The volcano map shows the DEGs of each two comparing groups (Figure 3B), in which 2347 up-regulated genes and 1472 down-regulated genes were found. To understand the main gene function and expression pattern change after TGFβ signaling pathway inhibition, the up-regulated genes, and down-regulated genes were analyzed by GO enrichment, respectively (Supplemental Table S4). The results showed that the up-regulated pathways were mainly enriched in the ion transport, channel activity, lipid biosynthesis, and glutathione catabolic processes (Figure 3C), while the down-regulated pathways were enriched in the catabolism pathway, lipid transport, microtube-based process, and extracellular region (Figure 3E). Among the total of 3819 DEGs (Supplemental Table S5), *ZBED5*, *ZIC4*, *LACTBL1*, *WNT3A*, and *B4GALT1* were at the top of the up-regulated gene list (Figure 3D), and *HSDL1*, *CCDC172*, *MSANTD3*, *RSPO2*, and *FAT4* showed the opposite tendency (Figure 3F). For example, *FAT4* is a member of the protocadherin family, which is a key factor in polarity establishment and signal transduction [39], reflecting a relatively strong function in cell–cell communication and polarity rearrangement in tail regression.

**Figure 4:**
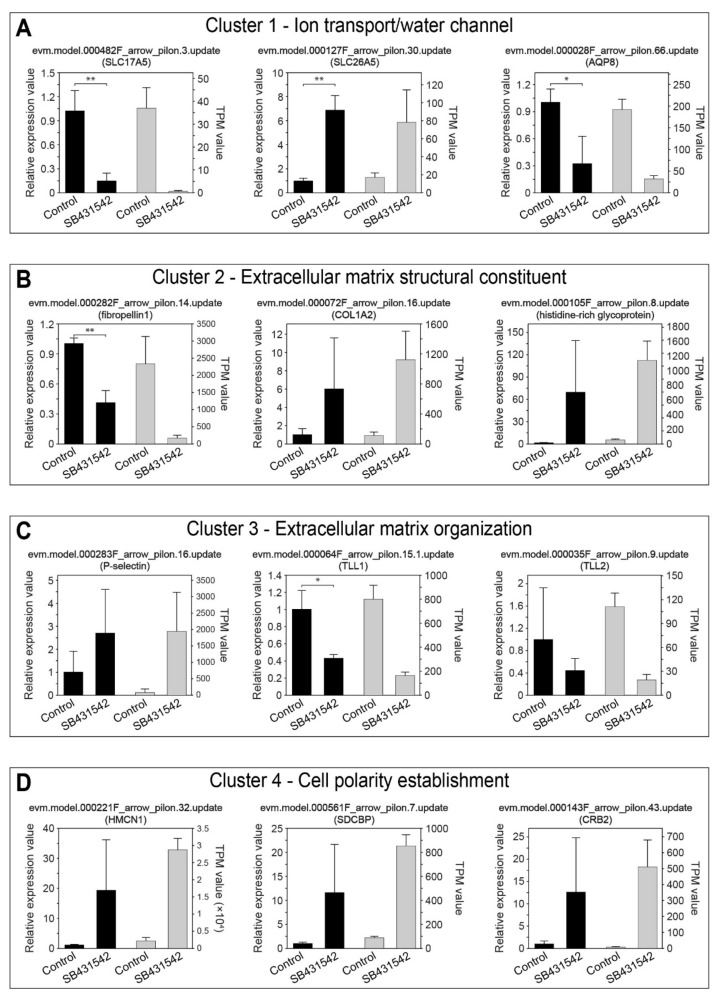
qRT-PCR verification of the key downstream genes of the TGFβ signaling pathway for four main functional categories, including ion transport and water channel (**A**), extracellular matrix structural constituent (**B**), extracellular matrix organization (**C**), and cell polarity establishment (**D**). Subtitles represent the gene ID and gene functional annotation by similarity comparison. Black and gray boxes represent the qRT-PCR results and transcriptome data (TPM value) of the genes, respectively. Mann–Whitney *U*-test and Student’s *t*-test are performed on qRT-PCR data, depending on whether data show normality distribution. * *p* < 0.05; ** *p* < 0.01.

To examine the gene expression level after the TGFβ signaling pathway inhibition, quantitative Real-Time PCR (qRT-PCR) was performed to try to further understand the downstream functional gene regulation in tail regression. According to the main GO enrichment results, together with differentially expressed level (log2FoldChange) and transcript abundance (TPM value), we manually selected 12 genes belonging to four main functional categories, which are ion transporters/water channel, extracellular matrix structural constituent, extracellular matrix organization, and cell polarity establishment. The three representative genes selected for ion transporters/water channel cluster were solute carrier family 17 member 5 [*SLC17A5*], solute carrier family 26 member 5 [*SLC26A5*, *prestin*], and aquaporin 8 [*AQP8*] (Figure 4A). SLC26A5 has been reported to be involved in notochord lumen formation in the *C. robusta* tail elongation process [36,40], while aquaporin has been known to regulate cell volume [41]; thus, ion transporters/water channel gene cluster is an ideal candidate in controlling tissue volume change during rapid tail regression process with drastic shape changes and tissue remodeling. The selected genes for extracellular matrix structural constituent and extracellular matrix organization clusters are *fibropellin1*, collagen type I alpha 2 chain [*COL1A2*], and *histidine-rich glycoprotein*, and *p-selectin*, tolloid-like 1 [*TLL1*], and tolloid-like 2 [*TLL2*], respectively (Figure 4B,C). Collagen, an essential extracellular matrix structural component, has been reported to coordinate multi-tissue elongation in ascidian [42], and showed a down-regulated pattern in *C. robusta* in swimming larva stage to tail regression stage transition [43]. The up-regulation of *COL1A2* after SB431542 treatment might partly explain the failure of tail regression. Meanwhile, two metalloproteinase family proteins, *TLL1* and *TLL2*, all showed down-regulated expression, further validating the hypothesis that the dysfunction of extracellular matrix remodeling may lead to tail regression abnormity. The last cluster is related to cytoskeleton rearrangement, including Hemicentin1 [*HMCN1*], syndecan binding protein [*SDCBP*], and crumbs cell polarity complex component 2 [*CRB2*]. Previous studies have shown that actomyosin contractility is necessary for tail regression and epidermis invagination [11]; although in this study we did not directly find out the expression level change in any cytoskeleton component or motor proteins, we still detected several cell polarity-related genes. For example, *HMCN1* is involved in the TGFβ-mediated rearrangement of the podocyte cytoskeleton and cleavage furrow maturation during cytokinesis [44,45], and thus may help regulate actomyosin cortex to generate contractility force to drive tail regression.

To sum up, we performed RNA-seq and comparative analysis for the TGFβ pathway inhibition. The transcriptomic profile showed that four gene categories are mainly enriched in the presence of TGFβ pathway inhibitor treatment, including ion transporters/water channel, extracellular matrix structural constituent, extracellular matrix organization, and cell polarity establishment. The gene expression level was verified by qRT-PCR experiment, and we identified that *SLC26A5*, *COL1A2*, *TLL*, and *HMCN1* are the potential TGFβ signaling pathway target genes in regulating tail regression.

### 3.5. Multi-Species Comparison Explains the Conservation and Divergence of Tail Regression in Ascidians

To investigate the conservation of gene regulation during the tail regression process in ascidians, transcriptome data from three developmental stages (27 hpf, 29 hpf, and 6 dpf, corresponding to hsl, trl, and mj, respectively) of *C. robusta* were selected, with three biological replicates per time point (Figure S7). A differential expression analysis was performed on the data from the three time points, and differentially expressed genes (DEGs) were identified between the time points (Figure S8).

**Figure 5:**
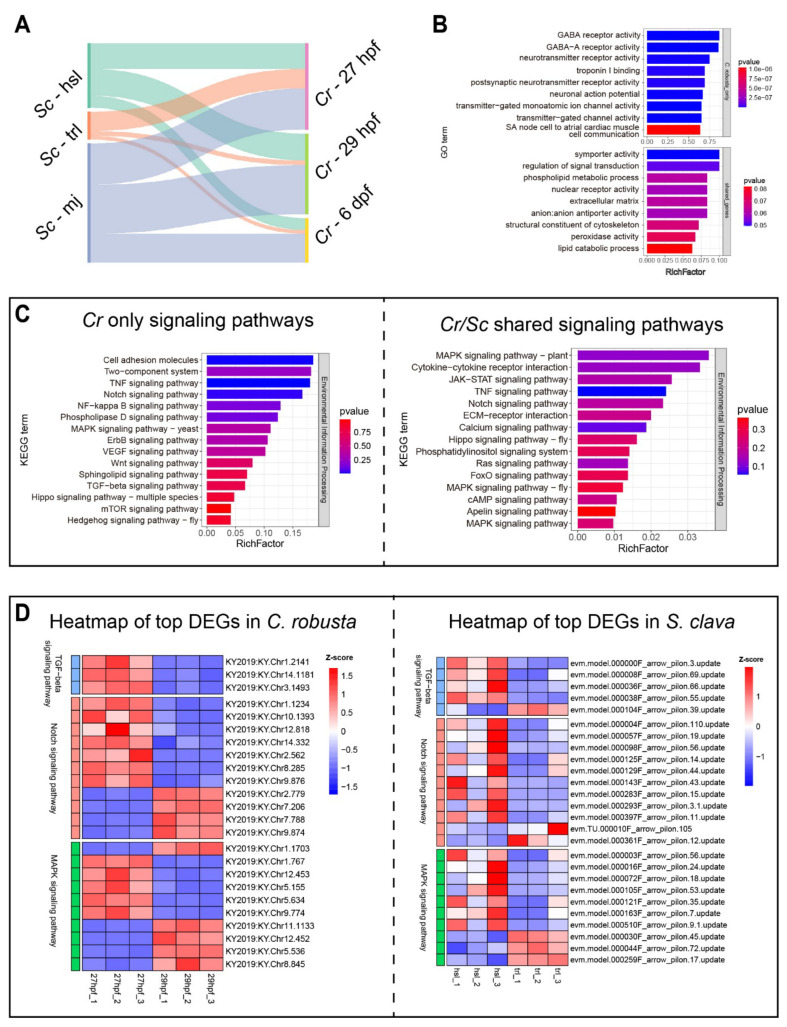
Conservation and divergence of gene regulation patterns during tail regression in ascidians. (**A**) Sankey plot showing the connectivity between three developmental stages of tail regression in *S. clava* and *C. robusta*. Cr-27hpf, 29hpf, and 5dpf represent the hsl, trl, and mj stages, respectively. (**B**) GO enrichment analysis for *Cr/Sc*-shared or *Cr*-only genes. (**C**) Signal transduction pathways enrichment for *Cr/Sc*-shared or *Cr*-only genes by KEGG analysis. (**D**) Heatmap of the top DEGs in the TGFβ pathway, Notch pathway, and MAPK pathway in *C. robusta* (left) and *S. clava* (right).

A homologous gene set between *C. robusta* and *S. clava* was established to help assess the similarity in gene expression. A total of 8089 homologous genes are identified. Next, we used RNentropy to calculate the stage-specific expression genes for the three time points in *S. clava* and *C. robusta* (Supplemental Table S6). Homologous genes that are specifically expressed at each time point were then extracted to characterize the similarity of the tail regression stages at different time points (Figure 5A). The results showed that the tail regression process in *S. clava* and *C. robusta* does not align well in terms of stage correspondence. Furthermore, the similarity between the three time points of *S. clava* and the first two time points of *C. robusta* is higher, which may suggest some differences in the tail regression processes between the two species.

We then focused on two key stages of tail regression, hsl vs. trl, extracting differentially expressed homologous genes from the homologous gene set and performing GO and KEGG enrichment analyses (Supplemental Tables S7 and S8). The results showed that the tail regression process in *C. robusta* is concentrated more into neuro-related pathways, such as GABA receptor activity and neurotransmitter receptor activity, involved in the regulation of postsynaptic membrane potential (Figure 5B), which differs significantly from *S. clava* (Figure 1C). However, the differentially expressed genes in both species are commonly involved in pathways like symporter activity, regulation of signal transduction, and phospholipid metabolic processes (Figure 5C). This suggested that unlike *S. clava*, *C. robusta* involves significant neuro-related transitions during the tail regression phase. Furthermore, we presented a heatmap of the top differentially expressed genes in three significant signaling pathways that are already known to participate in *C. robusta* embryogenesis or studied in this research (Figure 5D and Supplemental Table S9). In the TGFβ pathway, most of the differentially expressed genes were significantly down-regulated. In contrast, in the Notch and MAPK pathways, there were both up-regulated and down-regulated genes. However, in *S. clava*, these pathways showed a predominance of significantly down-regulated genes, which differs from *C. robusta*.

To summarize, we identified the conservation and divergence of the mechanism of tail regression between the model organism *C. robusta*, and *S. clava*. They both needed the regulation of signal transduction, but were varied in the detailed signal transduction pathways. Meanwhile, gene regulation in *C. robusta* tail regression is more involved in neuro-related pathways, while in *S. clava* it is more involved in other cellular processes like ion transport, cytoskeleton remodeling, and cell–matrix adhesion.

## 4. Discussion

The tail regression during the metamorphic development of ascidians has gradually become an emerging model for studying organ morphogenesis and pattern formation in recent years. Ascidians exhibit rapid embryonic development, and the process of tail regression occurs swiftly, taking only tens of minutes to a few hours [12,21]. In comparison, the typical time scale for tail disappearance in the classic chordate model for studying metamorphic development, *Xenopus laevis*, is around several days [46]. Therefore, the tail regression process in ascidians may involve more dramatic morphological changes, more precisely gene expression regulation, and more coordinated multi-tissue movements. Additionally, the regressed tail tissue in ascidians will partially undergo reprogramming and trans-differentiation into adult tissues or repurpose through apoptosis for energy reuse. This highly efficient and economical regression mechanism provides a valuable framework for studying the ecological and evolutionary significance of metamorphic development. Studies have shown that during tail regression in *C. robusta*, the outer epidermal tissue shortens and invaginates via pMRLC-mediated cortical contraction, while the inner tissues become coiled and move into the trunk [11]. However, the tissue structure of the tail-regressed larva in *S. clava* is entirely different from that of *Ciona*, and the regression occurs within a much shorter time frame [12], suggesting the presence of novel and more efficient mechanisms driving tail regression. In this study, the transcriptomic and GO enrichment analyses of different developmental stages before and after tail regression in *S. clava* revealed that the differentially expressed genes were mainly enriched in pathways such as tricarboxylic acid cycle, regulation of signal transduction, phospholipid transport, and ATP-dependent microtubule motor activity (Figure 1C), indicating that cellular energy metabolism and cytoskeletal regulation may play dominant roles.

The transition of developmental processes regulated by upstream signals is fundamental to all animals undergoing metamorphosis. Previous studies have primarily focused on hormone-dependent regulatory mechanisms. For example, in *X. laevis*, thyroid hormone is considered the most critical driving signal for tail resorption [47]. By regulating tissue sensitivity to thyroid hormone signals through tissue-specific thyroid hormone receptor expression, it can lead to tail resorption via direct TH-responsive cell death and cell death caused by the degradation of the extracellular matrix [46,48]. In vertebrate development that undergoes rapid maturation and environmental adaptation, such as the perinatal stages of mammals and the hatching of birds, hormonal regulation also plays a key role [49,50]. In ascidian model organisms like *C. robusta* and in invertebrates with lower evolutionary status such as mollusks and echinoderms, the role of thyroid hormones remains controversial. Current research suggests that the GABA pathway contributes significantly to the metamorphic transition from swimming larvae to sessile adults [9,51,52]. In this study, we explored the functional role of signal transduction pathways in ascidian metamorphosis. The KEGG enrichment analysis and experimental validation revealed that the inhibition of the TGFβ pathway leads to abnormal tail regression (Figure 2). This finding suggests that the TGFβ pathway may function as a parallel mechanism to classical neurotransmitter and hormonal regulation in controlling ascidian metamorphosis.

Through comparative transcriptome analysis and qRT-PCR verification after TGFβ pathway inhibition, we identified four key functional groups of downstream target genes: ion transporters/water channel, extracellular matrix structural constituent, extracellular matrix organization, and cell polarity establishment (Figure 3 and Figure 4). In *X. laevis*, tail shortening primarily depends on MMP-induced ECM degeneration-related cell death and Caspase-dependent cell apoptosis driven by thyroid hormone [46], along with the immune-mediated clearance of apoptotic cells [53]. In contrast, studies have shown that the direct driving forces of tail shortening in ascidians are cortical contraction and tissue rearrangement [11], with most regressed tail cells still remaining viable [54]. The cell polarity protein HMCN1, identified in this study, may act as a cytoskeletal regulator driving the active contraction of the tail. Additionally, several extracellular matrix components and regulatory proteins, commonly known as the downstream targets of the TGFβ pathway, were significantly enriched and differentially expressed, suggesting their possible involvement in the rapid tail regression process. Furthermore, numerous ion transport-related genes, such as SLC26A5, were up-regulated following the TGFβ pathway inhibitor treatment, indicating that osmotic homeostasis and cell volume regulation might also be crucial factors in tail regression.

In conclusion, RNA sequencing on the different developmental stages in *S. clava* provided a detailed profile of the gene regulation in tail regression. By performing the KEGG enrichment analysis and inhibitor treatment experiment verification, the TGFβ signaling pathway was proved to be necessary for the stop of larvae swimming and transition to sessile tail-regressed larvae. To further understand the downstream gene regulation of the TGFβ signaling pathway in tail regression, a comparative analysis for the TGFβ pathway inhibition was performed. Together with qRT-PCR verification, we identified four critical gene functional categories, including ion transporters/water channel, extracellular matrix structural constituent, extracellular matrix organization, and cell polarity establishment. A cross-species transcriptional profile between two typical species in the ascidian, *C. robusta* and *S. clava*, indicates the similarity and difference in gene regulation in tail regression. The result showed that gene regulation in *C. robusta* is more involved in neuro-related pathways, while in *S. clava* it is more involved in other cellular processes like ion transport, cytoskeleton remodeling, and cell–matrix adhesion.

## Figures and Tables

**Table 1:** Primer sequences used in the qRT-PCR.

Gene Name	Forward Primer (5′-3′)	Reverse Primer (5′-3′)
SLC26A5	AAAGCAACGCCAACAGAGG	TCATGTCCAAGACGAAATGAGTAA
SLC17A5	ACTGGCGGATTGCCTCATC	TGGCTGCTGGTACACTTGGTC
AQP8	TTTCGGTCCAGCGGTTGT	ATCTAATGGTCCTTCTCCATCGT
fibropellin1	TATTGTCAGTGCGACAGAGGTG	GACATTTTGCGTGGGGATT
COL1A2	TGTAAACGGAACCAATGGAATG	GCTGACTGTTGTAATCGGCACT
CRB2	CCCGAATACGGAAATCGAGA	CCGAGGGCAAATGTCAGAAC
HMCN1	GACTCGCACCCGTAAGTGTTT	ACGCTGCATTCGCTCCAT
SDCBP	AGATAGTAGCGGCCATGTTGG	CGCATTGTCCGTTCACTTCAC
TLL1	CGCGGAAACGCTGTTAGG	CACGGTGGTGATCTTTGTGG
TLL2	AAGCAGTACGAGGGGAAGATTACAT	CTGTTGATGCAGCCGGTGTAA
glycoprotein	ATCACCACCATCACCATGGACC	GTGTGGATATCCTCCGTGACCTG
P-selectin	GAAGCACTGAGATCCCAAGGAGTTCT	GTCGCAAGGATCGCCACAAATATTT
β-actin	AATCGTGACCAACTGGGATG	GCTGGAGTATTGAAGGTTTCGA

## Data Availability

(1) The transcriptome data of *S. clava* used for expression analysis were deposited in the NCBI SRA database (accession number PRJNA1232216). (2) Any information required to reanalyze the data reported in this paper is available from the lead contact upon request.

## Supplementary Materials

The following supporting information can be downloaded at https://www.mdpi.com/article/10.3390/cells14070546/s1, Figure S1: RNA-seq data for nine samples from three developmental stages of S. clava with three biological repeats quality visualization; Figure S2: Heat map diagram of the top DEGs expression level for four main functional categories; Figure S3: KEGG pathway enrichment of the DEGs in different stages; Figure S4: Quantification of the proportion of tail regression; Figure S5: Experimental procedure on inhibitor treatment experiment and RNA sequencing samples preparation; Figure S6: RNA-seq data for SB431542 inhibitor treatment samples quality visualization; Figure S7: RNA-seq data for nine samples from three developmental stages of *C. robusta* with three biological repeats quality visualization; Figure S8: Volcano map of DEGs in three developmental stages of *C. robusta*. Table S1: GO enrichment of 3 stages in *S. clava*; Table S2: KEGG enrichment of 3 stages in *S. clava*; Table S3: DEGs in the SB431542 inhibitor treatment and control groups; Table S4: GO enrichment of DEGs in the SB431542 inhibitor treatment and control groups; Table S5: Gene expression profile of DEGs in the SB431542 inhibitor treatment and control groups; Table S6: Homologous genes of 3 stages between *C. robusta* and *S. clava*; Table S7: GO enrichment of *C. robusta* only and shared genes; Table S8: KEGG enrichment of *C. robusta* only and shared genes; Table S9: Gene expression profile *of C. robusta* and *S. clava* in different pathways.
