# Supplementary material for: Transcriptome Analysis Reveals the Requirement of the TGFβ Pathway in Ascidian Tail Regression
Source: Cells. 2025 Apr 4;14(7):546. doi: 10.3390/cells14070546 (PMC11988888; doi:10.3390/cells14070546)
Supplement: Supplementary file 1 [file cells-14-00546-s001.zip › Figures S1-S8.pdf]

Supplemental information for

Transcriptome Analysis Reveals the Requirement of the TGFβ Pathway in Ascidian Tail Regression

Wenjie Shi [1,†], Penghui Liu [1,†], Dongyu Yang [1], Yuan Zhuang [1], Boyan Lin [1] and Bo Dong [1,2,3,*]

[1] Fang Zongxi Center for Marine EvoDevo, MoE Key Laboratory of Marine Genetics and Breeding, College of Marine Life Sciences, Ocean University of China, Qingdao 266003, China; wenjie.shi0713@stu.ouc.edu.cn (W.S.); linboyan@stu.ouc.edu.cn (B.L.)liupenghui@stu.ouc.edu.cn (P.L.); yangdyu123456@163.com (D.Y.); zhuangyuanzty1313@163.com (Y.Z.)

[2] Laboratory for Marine Biology and Biotechnology, Qingdao National Laboratory for Marine Science and Technology, Qingdao 266237, China

[3] Institute of Evolution & Marine Biodiversity, Ocean University of China, Qingdao 266003, China

* Correspondence: bodong@ouc.edu.cn

† These authors contribute equally to this article.

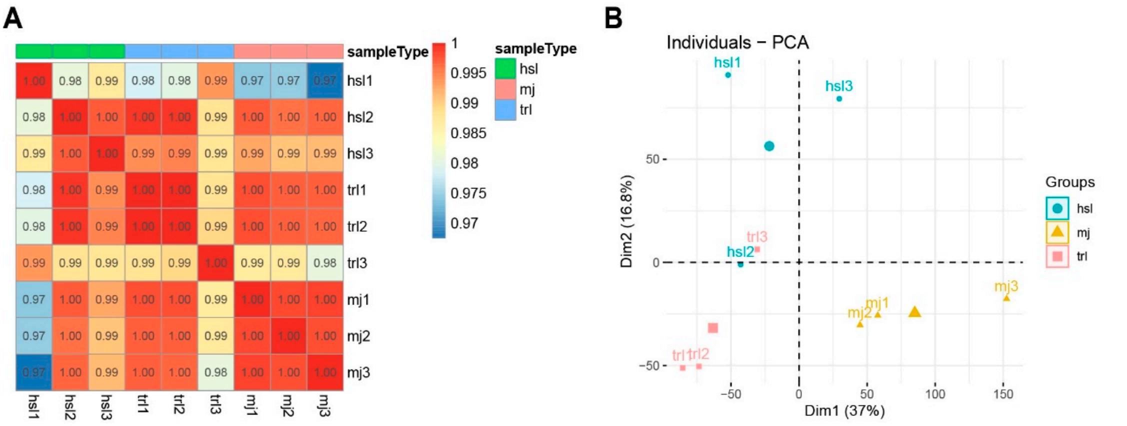

Figure S1. RNA-seq data for nine samples from three developmental stages of *S. clava* with three biological repeats quality visualization. (A) Correlation matrix of the nine samples. (B) Principal component analysis (PCA) of the nine samples.

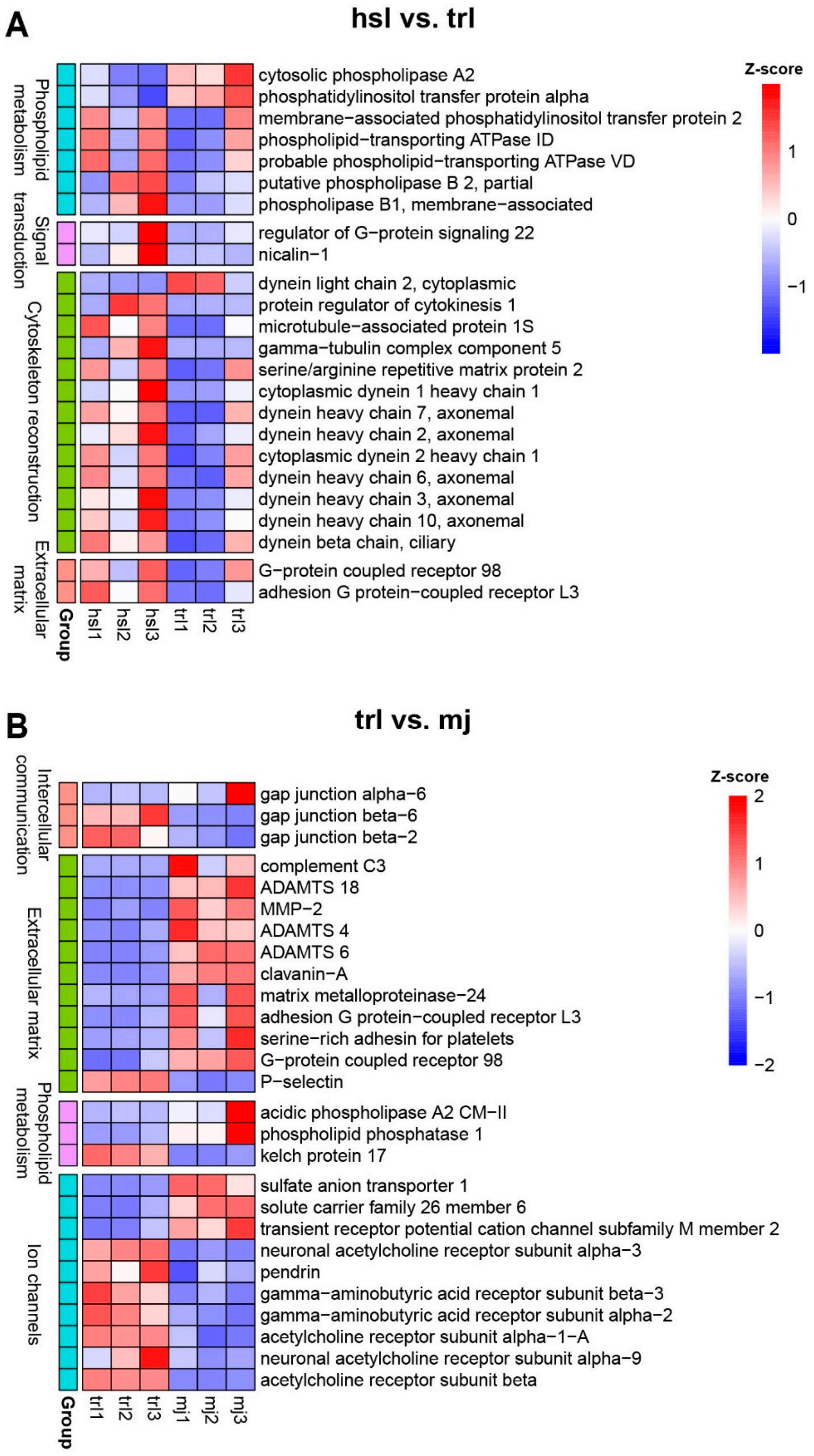

Figure S2. Heat map diagram of the top DEGs expression level for four main functional categories. (A) Top DEGs expression data for four categories in hsl vs. trl, including phospholipid metabolism, signal transduction, cytoskeletion reconstruction and extracellular matrix. (B) Top DEGs expression data for four categories in trl vs. mj, including intercellular communication, extracellular matrix, phospholipid metabolism and ion channels. Color indicates the relative expression level.

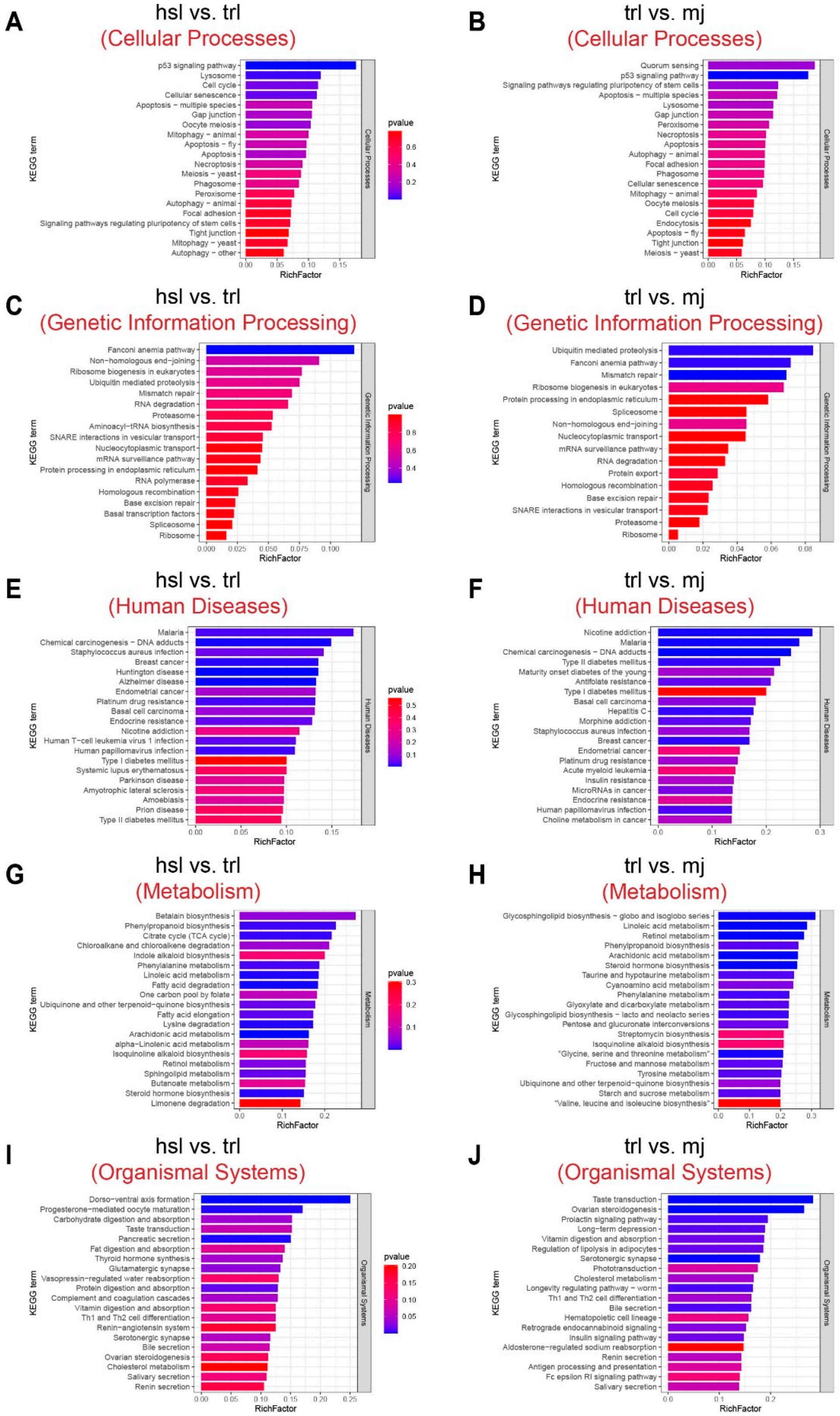

Figure S3. KEGG pathway enrichment of the DEGs in different stages showing the potential involved cellular processes (A-B), genetic information processing (C-D), related human dieases (E-F), metabolism (G-H), and organismal systems (I-J).

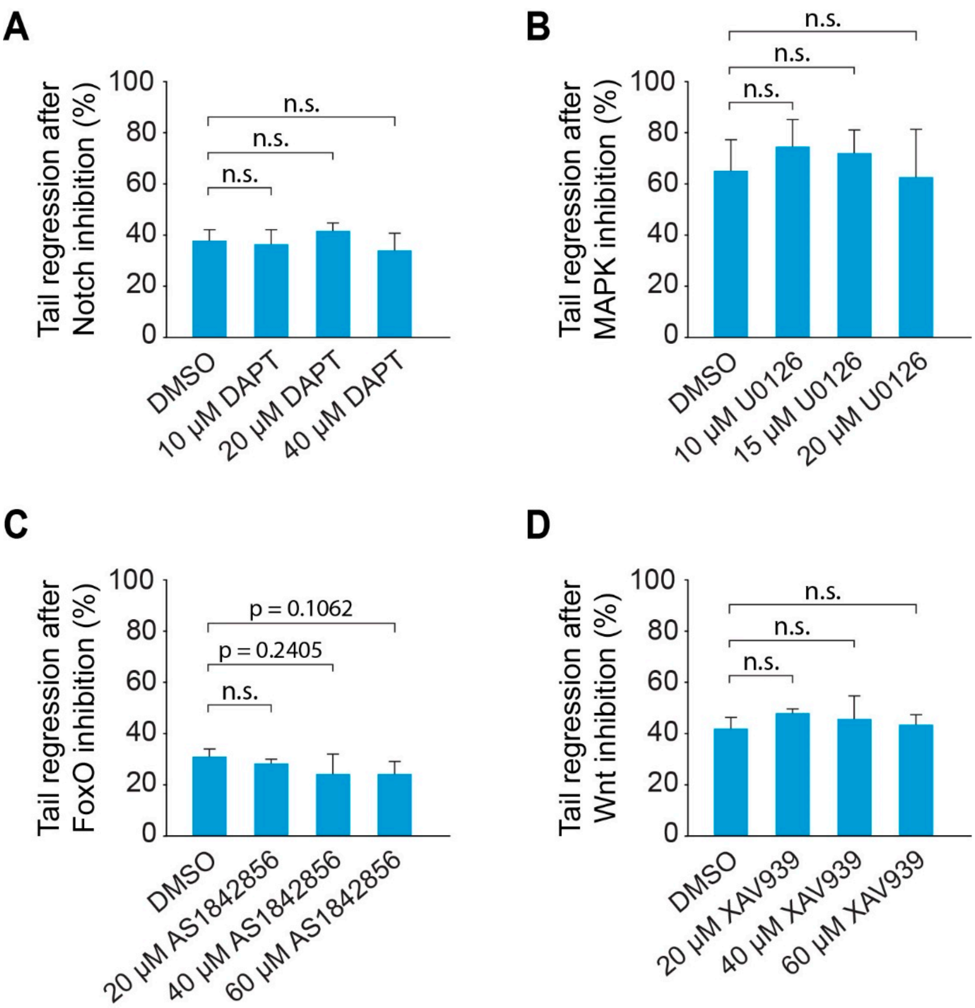

Figure S4. Quantification of the proportion of tail regression with Notch signaling pathway (A), MAPK signaling pathway (B), FoxO signaling pathway (C), and Wnt signaling pathway (D) inhibitor treatment (N = 3 embryo batches). Mann–Whitney *U*-test and student *t*-test are performed, depending on whether data shows normality distribution. n.s., no significant difference.

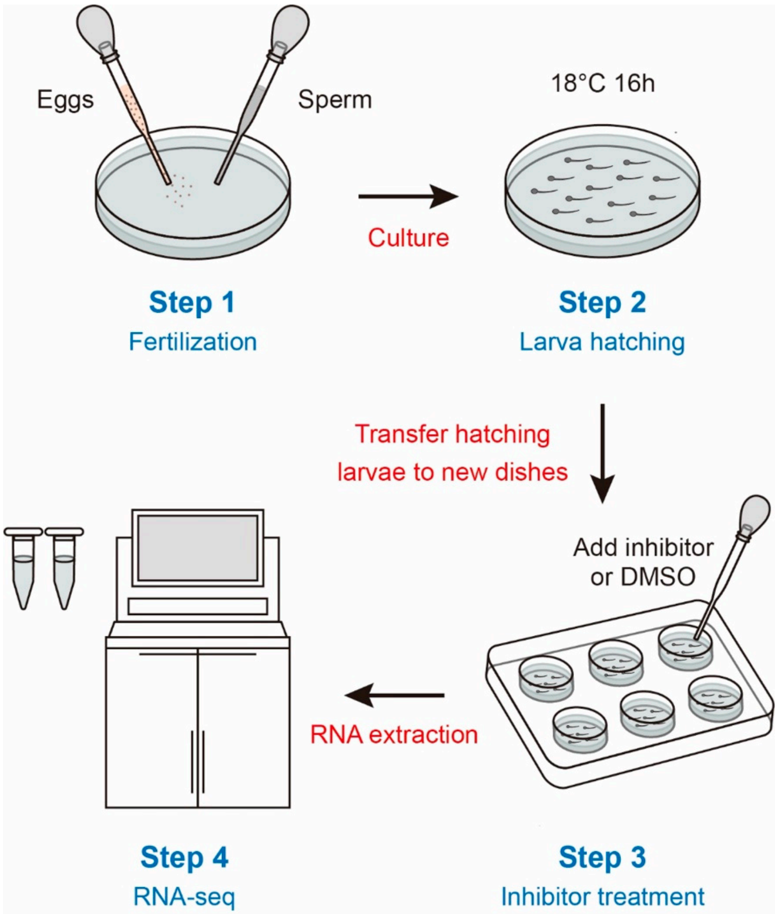

Figure S5. Experimental procedure on inhibitor treatment experiment and RNA sequencing samples preparation. Step 1, collect mature eggs and sperm from *S. clava* adults respectively, and mix the two to obtain fertilized eggs. Step 2, incubate the fertilized eggs in FSW at 18 °C for 16 hours to hatching. Step 3, select hatched larvae to new dishes (200 larvae for each dish), add inhibitor or DMSO into the dish, culture at 23 °C for around 3 hours till most larvae in DMSO treatment group have initiated tail regression process. Step 4, collect inhibitor or DMSO treatment larvae samples, extract RNA and perform RNA sequencing. Repeat step 3 and 4 for four times to have four biological repeats. See detailed procedures and methods in Materials and Methods.

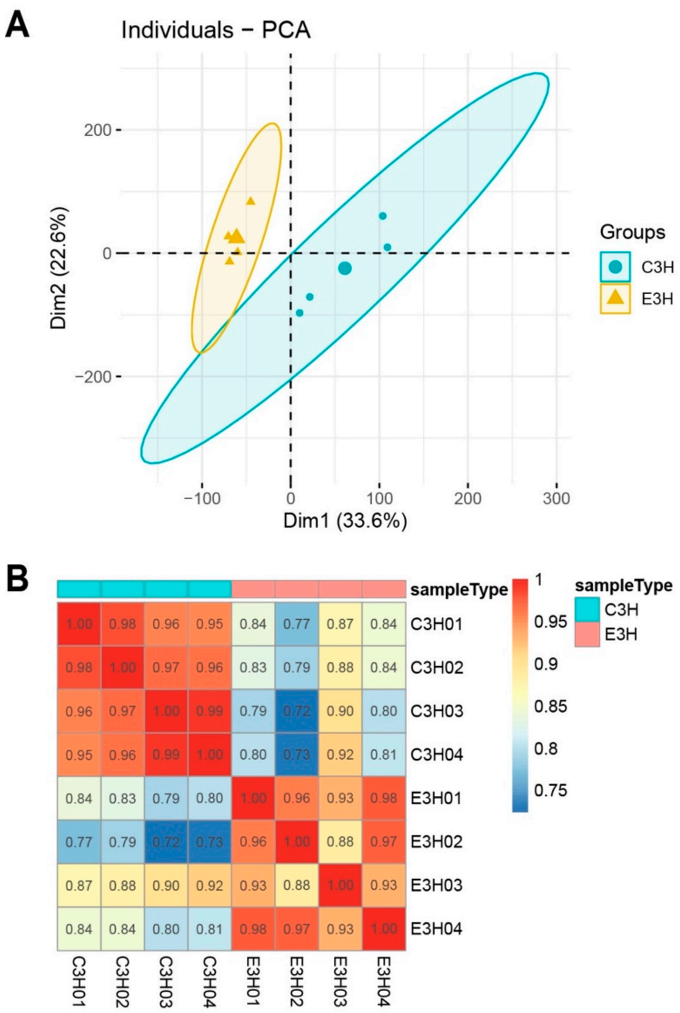

Figure S6. RNA-seq data for SB431542 inhibitor treatment samples quality visualization. (A) Principal component analysis of the four biological repeats of SB431542 and DMSO treatment RNA-seq data. (B) Correlation matrix of SB431542 and DMSO treatment RNA-seq data. C3H, control group (DMSO treatment) at tail regression stage (around 3 hph), E3H, experiment group (SB431542 treatment) at tail regression stage.

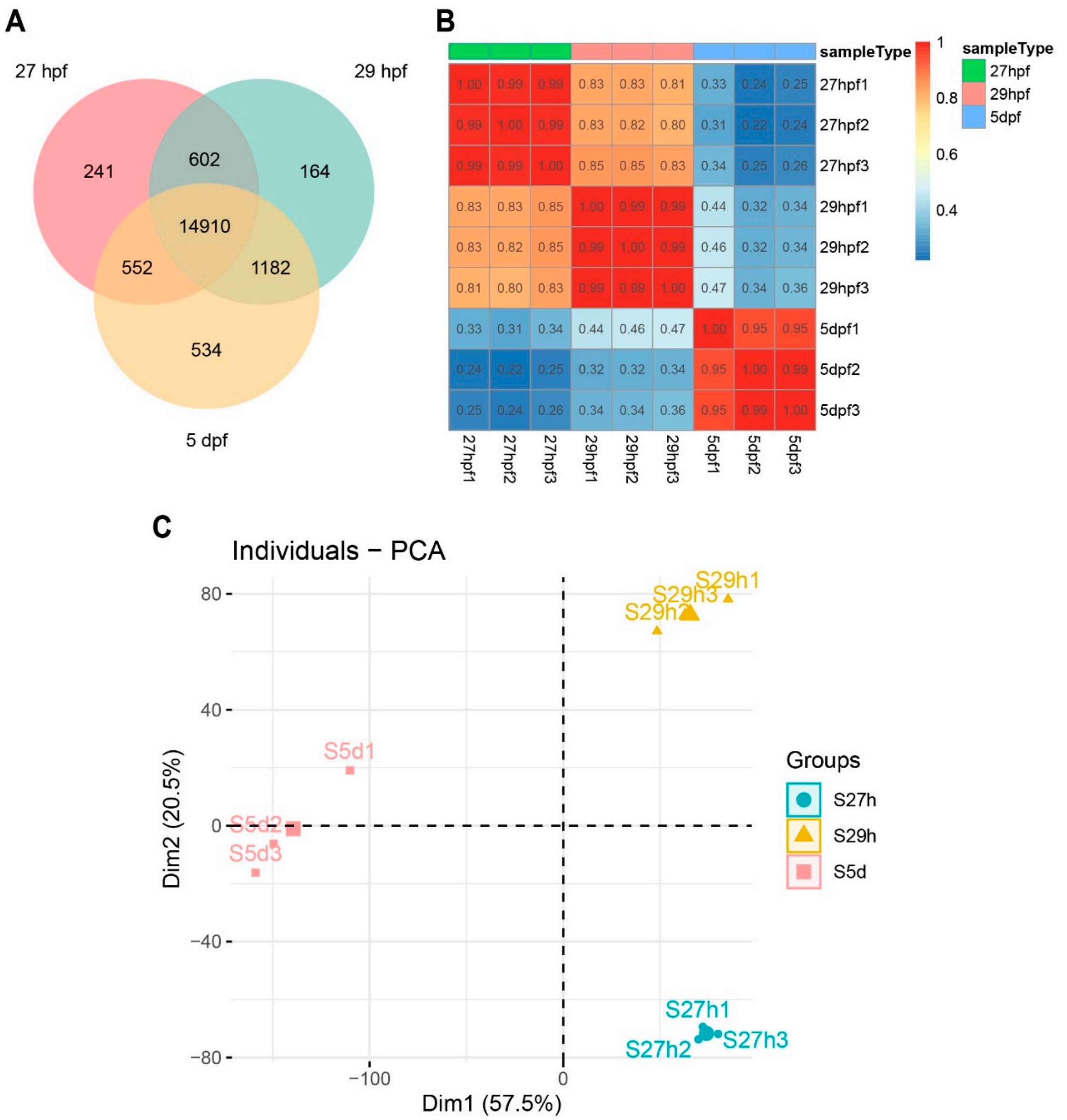

Figure S7. RNA-seq data for nine samples from three developmental stages of *C. robusta* with three biological repeats quality visualization. (A) Venn diagram showing the shared and unique expressed genes in 27 hpf (correspond to hsl), 29 hpf (trl), and 5 dpf (mj) of *C. robusta*, respectively. (B) Correlation matrix of the nine samples. (C) Principal component analysis (PCA) of the nine samples.

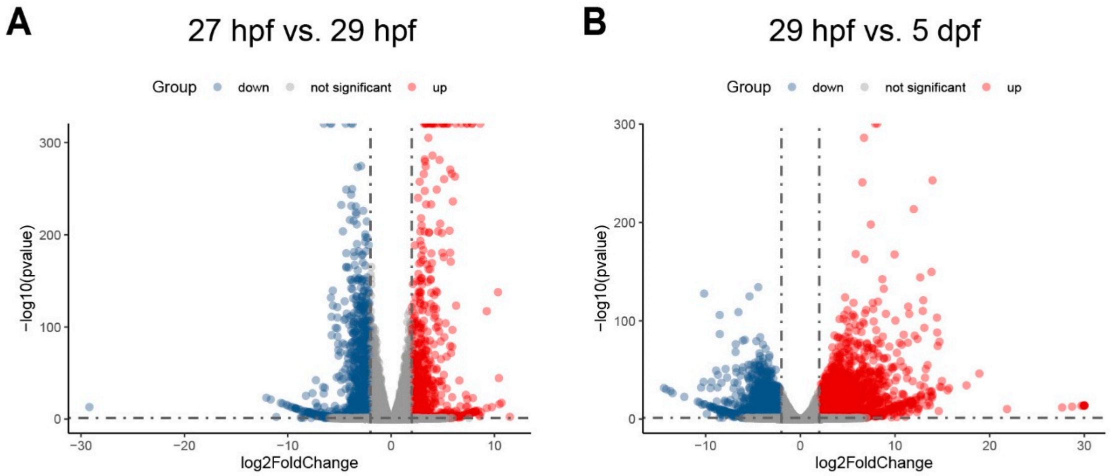

Figure S8. Volcano map of DEGs in three developmental stages of *C. robusta*, including 27 hpf vs. 29 hpf (A) and 29 hpf vs. 5 dpf (B).
